# Supplementary material for: Selective nutrient incorporation may underestimate heterotrophy of a mixotrophic reef-building coral
Source: Commun Biol. 2025 Aug 26;8:1285. doi: 10.1038/s42003-025-08621-8 (PMC12381372; doi:10.1038/s42003-025-08621-8)
Supplement: Supplementary file 2 — Supplemental Material [file 42003_2025_8621_MOESM2_ESM.pdf]

Supporting Information

treatment	tank replicate	cumulative nauplii capture	cumulative biomass capture	mean treatment biomass capture	std. deviation biomass capture
**Control**	A	0	0.00	0.00	0.00
**Control**	B	0	0.00		
**Control**	C	0	0.00		
**F_2x**	A	728	2.33	3.11	0.83
**F_2x**	B	1244	3.98		
**F_2x**	C	948	3.03		
**F_6x**	A	2195	7.02	6.61	1.60
**F_6x**	B	1516	4.85		
**F_6x**	C	2490	7.97		
**B_F_6x**	A	1440	4.61	5.36	1.05
**B_F_6x**	B	1536	4.92		
**B_F_6x**	C	2052	6.57		

Supplementary Table 1. Cumulative nauplii and biomass capture over duration of experiment. Raw data for each tank in each condition provided, as well as mean and standard deviation of cumulative biomass capture for each treatment group.

	control	F_2x	F_2x	F_2x	F_2x	F_6x	F_6x	F_6x	F_6x	B_F_6x	B_F_6x	B_F_6x	B_F_6x
	**mean ±sd**	**mean ±sd**	**estimate**	**statistic**	**p value**	**mean ±sd**	**estimate**	**statistic**	**p value**	**mean ±sd**	**estimate**	**statistic**	**p value**
**host FA ($\mu g\ cm^{-2}$)**	0.08 ±0.05	0.1 ±0.05	0.01	0.63	0.55	0.08 ±0.03	0.00	0.14	0.89	0.02 ±0.01	-0.06	-3.77	0.00
**host protein ($mg\ cm^{-2}$)**	0.86 ±0.35	1.14 ±0.69	0.25	1.07	0.32	1.1 ±0.52	0.29	1.25	0.25	0.63 ±0.29	-0.14	-0.60	0.57
**aragonite growth ($mg\ cm^{-2}\ d^{-1}$)**	0.88 ±0.53	1.66 ±0.94	0.74	2.92	0.02	1.64 ±0.65	0.73	2.94	0.03	0.1 ±0.56	-0.73	-2.95	0.03
**symbiont density ($10^6\ cm^{-2}$)**	0.45 ±0.32	0.68 ±0.51	0.18	1.27	0.24	0.67 ±0.43	0.24	1.74	0.12	0.05 ±0.08	-0.30	-2.08	0.07
**Chlorophyll a ($\mu g\ cm^{-2}$)**	13.54 ±5.2	20.42 ±10.08	6.22	3.33	0.02	26.87 ±10.22	13.93	7.68	0.00	2.47 ±1.93	-7.93	-4.06	0.01
**Chlorophyll c2 ($\mu g\ cm^{-2}$)**	4.82 ±3.96	8.65 ±7.07	4.04	2.06	0.08	8.92 ±5.88	4.00	2.07	0.08	2.61 ±2.66	-1.48	-0.76	0.47
**Chlorophyll total ($\mu g\ cm^{-2}$)**	18.36 ±7.02	29.08 ±14.82	10.42	2.96	0.02	35.79 ±13.72	17.96	5.20	0.00	5.08 ±4.1	-9.20	-2.54	0.04
**symbiont FA ($\mu g\ cm^{-2}$)**	69.06 ±11.13	70.4 ±15.47	5.88	1.57	0.12	67.65 ±13.99	0.14	0.04	0.97	NA	NA	NA	NA
**symbiont protein ($ng\ cell^{-1}$)**	0.5 ±0.35	0.65 ±1.2	0.27	1.24	0.22	0.47 ±0.37	0.11	0.52	0.61	NA	NA	NA	NA

Supplementary Table 2. Physiology metrics summary data (mean and standard deviation) with associated coefficient model estimates ('estimate'), F statistic ('statistic') and p value from linear mixed effects models. Host FA (B_F_6x: n = 29, control: n = 23, F_2x: n = 22, F_6x: n = 27), host protein (B_F_6x: n = 33, control: n = 31, F_2x: n = 27, F_6x: n = 33), aragonite growth (B_F_6x: n = 31, control: n = 27, F_2x: n = 25, F_6x: n = 32), symbiont density (B_F_6x: n = 33, control: n = 28, F_2x: n = 24, F_6x: n = 30), Chlorophyll a, c2 and total (B_F_6x: n = 33, control: n = 31, F_2x: n = 27, F_6x: n = 33), symbiont FA (control: n = 23, F_2x: n = 21, F_6x: n = 27), and symbiont protein (control: n = 28, F_2x: n = 24, F_6x: n = 31).

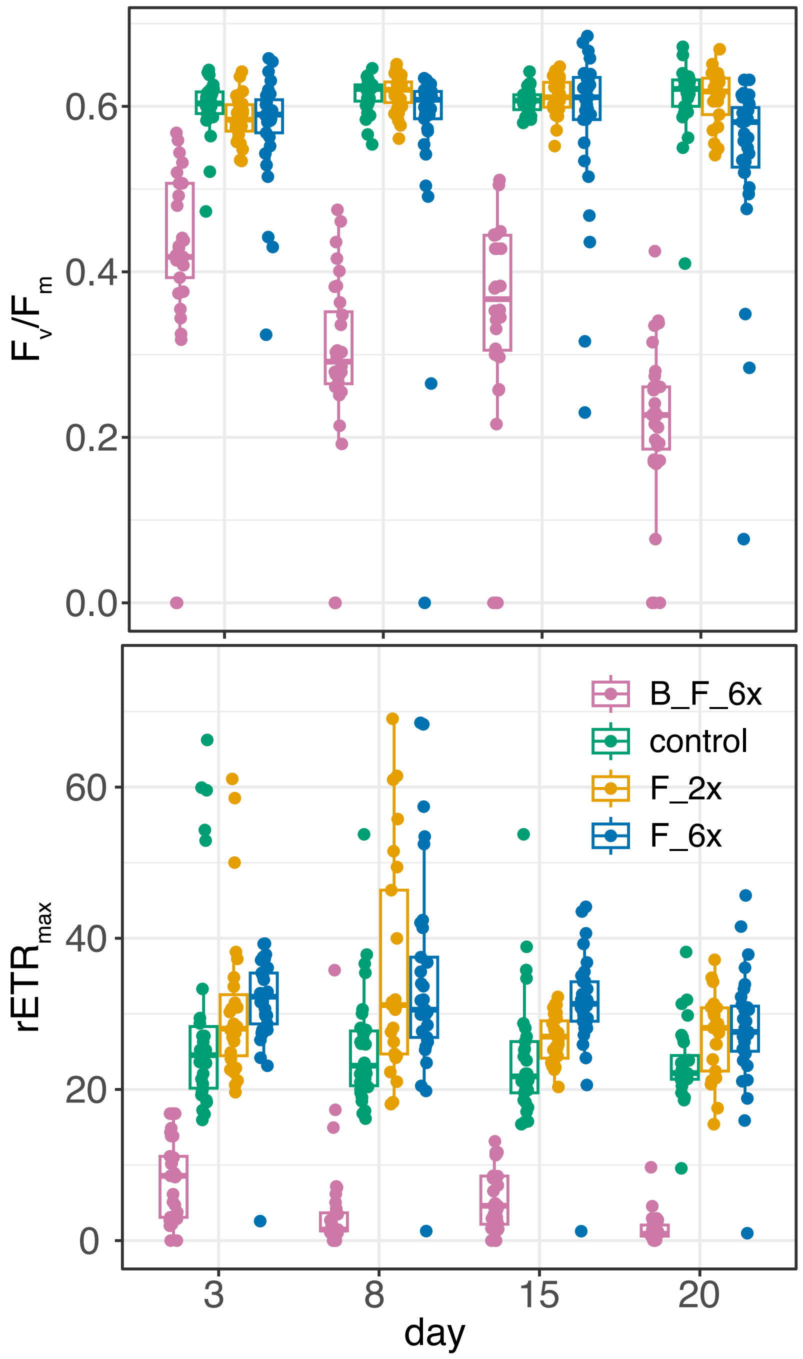

Supplementary Figure 1. Coral photosynthetic efficiency (F_v/F_m) and relative electron transport rate ($rETR_{max}$). Data are grouped by day of measurement and experimental treatment (B_F_6x: n = 32, control: n = 29, F_2x: n = 27, F_6x: n = 33). Box and whisker plots added on top of data points, horizontal lines in box represent median and quartiles while whiskers represent 1.5(IQR) distance from each upper and lower quartile.

	control	F_2x	F_6x	B_F_6x	day
**F_v/F_m day 4**	0.604 ±0.03	0.589 ±0.025	0.574 ±0.067	0.414 ±0.131	
**F_v/F_m day 9**	0.614 ±0.021	0.615 ±0.022	0.569 ±0.125	0.292 ±0.103	
**F_v/F_m day 16**	0.607 ±0.015	0.61 ±0.023	0.58 ±0.098	0.35 ±0.137	
**F_v/F_m day 21**	0.61 ±0.047	0.611 ±0.035	0.538 ±0.114	0.213 ±0.091	
**F_v/F_m estimate**		0.00	-0.03	-0.27	0.00
**F_v/F_m statistic**		-0.01	-1.28	-11.38	-4.26
**F_v/F_m p value**		0.99	0.23	0.00	0.00
**$rETR_{max}$ day 4**	23.5 ±5.2	27 ±5.7	27.8 ±8	1.6 ±1.9	
**$rETR_{max}$ day 9**	24.7 ±7.5	36.8 ±25	33.8 ±13.9	4.1 ±7.2	
**$rETR_{max}$ day 16**	24.3 ±8.5	26.8 ±2.9	30.6 ±7.3	5.7 ±4.1	
**$rETR_{max}$ day 21**	29.8 ±14.7	31.9 ±11.7	30.9 ±6.7	8.4 ±5.4	
**$rETR_{max}$ estimate**		6.00	5.54	-19.94	-0.32
**$rETR_{max}$ statistic**		2.84	2.66	-9.45	-4.63
**$rETR_{max}$ p value**		0.02	0.03	0.00	0.00

Supplementary Table 3. Photophysiology summary data (mean and standard deviation, B_F_6x: n = 32, control: n = 29, F_2x: n = 27, F_6x: n = 33) and model results grouped by day. Model results report coefficient model estimates (‘estimate’), F statistic (‘statistic’) and p value from linear mixed effects models.

	control	F_2x	F_2x	F_2x	F_2x	F_6x	F_6x	F_6x	F_6x	B_F_6x	B_F_6x	B_F_6x	B_F_6x
**biomarker**	**mean ±sd**	**mean ±sd**	**estimate**	**statistic**	**p value**	**mean ±sd**	**estimate**	**statistic**	**p value**	**mean ±sd**	**estimate**	**statistic**	**p value**
**C12:0**	0.1 ±0.1	0.2 ±0.1	-0.011	-0.204	0.838	0.3 ±0.3	0.082	1.574	0.118	0.3 ±0.2	0.139	2.548	0.012
**C14:0**	1.4 ±0.2	1.3 ±0.2	-0.086	-1.918	0.093	1.3 ±0.2	-0.043	-0.961	0.366	0.5 ±0.2	-0.922	-19.566	0.000
**C14:1**	0.1 ±0	0.1 ±0	-0.008	-0.989	0.325	0.1 ±0	0.010	1.203	0.231	0 ±0	-0.043	-5.235	0.000
**C16:0**	22.5 ±2	22.5 ±2.2	-0.082	-0.164	0.875	24 ±2	1.634	3.258	0.015	22.7 ±3	-0.107	-0.204	0.844
**C16:1n9**	0 ±0	0 ±0	-0.006	-0.208	0.836	0 ±0	-0.003	-0.123	0.903	0.1 ±0.2	0.041	1.383	0.169
**C16:1n7**	3.4 ±0.5	3.1 ±0.4	-0.141	-1.392	0.214	3.2 ±0.4	0.012	0.117	0.910	0.9 ±0.6	-2.391	-22.501	0.000
**C16:2**	0.1 ±0	0.1 ±0	-0.004	-0.217	0.834	0.1 ±0	0.022	1.242	0.253	0.1 ±0.1	-0.012	-0.675	0.519
**C18:0**	13.3 ±1.9	13.6 ±1.5	0.461	0.682	0.497	15.4 ±1.7	1.800	2.657	0.009	31.1 ±6.5	17.308	24.194	0.000
**C18:1n9**	10.5 ±1.6	9.7 ±1.6	-0.646	-1.118	0.296	8.4 ±2	-1.778	-3.073	0.015	4.6 ±1.4	-6.460	-10.975	0.000
**C18:1n7**	3.1 ±0.5	3.3 ±0.5	0.163	1.520	0.166	3.1 ±0.5	0.091	0.845	0.422	2.7 ±0.5	-0.438	-3.904	0.003
**C18:2n6**	0.8 ±0.1	0.9 ±0.1	0.145	2.472	0.040	1 ±0.1	0.236	4.001	0.004	1.5 ±0.4	0.723	11.875	0.000
**C18:3n6**	1.4 ±0.4	1.1 ±0.3	-0.263	-3.786	0.000	1 ±0.3	-0.389	-5.600	0.000	0.6 ±0.3	-0.689	-9.399	0.000
**C18:3n3**	0.1 ±0.1	0.6 ±0.1	0.412	4.020	0.005	0.5 ±0.1	0.337	3.289	0.013	0.9 ±0.5	0.858	8.203	0.000
**C18:4n3**	1.8 ±0.8	1.7 ±0.9	0.234	1.334	0.218	2 ±1.1	0.548	3.127	0.014	0.3 ±0.2	-0.973	-5.419	0.000
**C20:0**	0.7 ±0.1	0.6 ±0.1	-0.041	-1.096	0.314	0.7 ±0.1	-0.027	-0.727	0.494	1.1 ±0.3	0.389	9.892	0.000
**C20:1n9**	3.2 ±0.8	3 ±0.8	-0.090	-0.791	0.449	2.5 ±0.8	-0.533	-4.679	0.001	1.6 ±0.5	-1.789	-14.929	0.000
**C20:2**	0.7 ±0.1	0.8 ±0.3	0.073	1.631	0.106	0.7 ±0.1	-0.028	-0.624	0.534	1.1 ±0.2	0.288	6.172	0.000
**C20:3n6**	4.5 ±1	3.6 ±0.9	-0.908	-4.427	0.002	2.7 ±0.6	-1.798	-8.761	0.000	0.8 ±0.5	-3.813	-17.957	0.000
**C20:4n6**	8.6 ±1.5	9.5 ±1.4	0.534	1.259	0.244	8.9 ±1.4	-0.096	-0.226	0.827	12.1 ±2.4	3.975	9.134	0.000
**C20:4n3**	2.2 ±0.6	1.9 ±0.4	-0.207	-2.339	0.021	1.7 ±0.4	-0.380	-4.279	0.000	0.3 ±0.2	-1.810	-19.302	0.000
**C20:5n3**	2.1 ±0.4	2.7 ±0.4	0.556	4.132	0.003	3.4 ±0.7	1.197	8.884	0.000	3.1 ±1	1.183	8.376	0.000
**C22:0**	0.3 ±0.1	0.3 ±0.1	-0.012	-0.427	0.684	0.3 ±0.1	-0.007	-0.234	0.822	0.7 ±0.2	0.359	11.927	0.000
**C22:1n9**	0.7 ±0.1	0.7 ±0.2	-0.005	-0.097	0.925	0.6 ±0.1	-0.112	-2.105	0.066	0.6 ±0.2	-0.124	-2.292	0.047
**C23:0**	0.7 ±0.2	0.7 ±0.2	-0.042	-1.208	0.229	0.6 ±0.1	-0.172	-4.925	0.000	0.3 ±0.1	-0.445	-12.314	0.000
**C22:4n6**	6.2 ±1	6.7 ±1.1	0.375	1.199	0.265	7.1 ±1.1	0.684	2.182	0.061	9.2 ±1.9	3.204	9.916	0.000
**C22:5n3**	1.2 ±0.2	1.3 ±0.2	0.052	0.995	0.346	1.4 ±0.2	0.198	3.805	0.004	1.1 ±0.3	-0.111	-2.052	0.066
**C22:6n3**	10.3 ±1.7	9.9 ±1.7	-0.439	-1.280	0.234	8.9 ±1.7	-1.501	-4.370	0.002	1.8 ±1.3	-8.336	-23.100	0.000
**^{15}N (‰)**	-0.35 ±0.36	1.73 ±0.77	2.076	9.813	0.000	3.39 ±0.63	3.740	17.955	0.000	3.32 ±0.76	3.669	17.342	0.000
**^{13}C (‰)**	-17.04 ±0.98	- 17.51 ±1.27	-0.455	-1.593	0.147	- 17.46 ±0.8	-0.389	-1.387	0.203	-17.45 ±1.15	-0.391	-1.347	0.211
**C:N**	5.06 ±0.16	4.89 ±0.16	-0.174	-3.335	0.012	4.76 ±0.22	-0.303	-5.884	0.001	4.41 ±0.15	-0.675	-12.665	0.000

Supplementary Table 4. Host biomarker data summary and mixed effects linear model results. Table shows fatty acid (% total), isotope (permill) and elemental ratio summary data (mean and standard deviation, control: n = 31, F_2x: n = 27, F_6x: n = 32, B_F_6x: n = 33) with associated coefficient model estimates (‘estimate’), F statistic (‘statistic’) and p value from linear mixed effects models.

	control	F_2x	F_2x	F_2x	F_2x	F_6x	F_6x	F_6x	F_6x
**biomarker**	**mean ±sd**	**mean ±sd**	**estimate**	**statistic**	**p value**	**mean ±sd**	**estimate**	**statistic**	**p value**
**C12:0**	0.3 ±0.3	0.2 ±0.2	-0.020	-0.756	0.478	0.2 ±0.1	-0.037	-1.386	0.219
**C14:0**	5.4 ±1.2	4.5 ±0.9	-0.654	-3.361	0.012	4.2 ±0.7	-0.797	-4.128	0.005
**C14:1**	0.5 ±0.1	0.6 ±0.1	0.092	2.561	0.041	0.7 ±0.1	0.158	4.444	0.004
**C16:0**	23.1 ±2	21.8 ±1.7	-1.066	-1.594	0.160	21.6 ±2.9	-1.373	-2.065	0.084
**C16:1n9**	0.9 ±0.5	1 ±0.4	-0.011	-0.085	0.935	1.4 ±0.6	0.472	3.764	0.013
**C16:1n7**	7.4 ±1.7	6.1 ±1.1	-0.725	-2.846	0.024	5.8 ±1	-0.771	-3.049	0.019
**C16:2**	0.6 ±0.2	0.4 ±0.2	-0.089	-2.075	0.081	0.5 ±0.2	0.031	0.737	0.489
**C18:0**	6.4 ±1.5	6.3 ±1.2	-0.613	-1.893	0.107	6 ±1.2	-0.898	-2.802	0.032
**C18:1n9**	7.2 ±2	6.1 ±1.5	-0.911	-1.765	0.130	5.5 ±2.3	-1.844	-3.581	0.013
**C18:1n7**	1.2 ±0.4	1.5 ±0.4	0.164	2.382	0.020	1.4 ±0.3	0.067	0.982	0.329
**C18:2n6**	1.6 ±0.2	1.4 ±0.2	-0.062	-1.088	0.317	1.4 ±0.2	-0.183	-3.261	0.018
**C18:3n6**	5.5 ±1.2	3.8 ±0.9	-1.418	-4.877	0.002	3.4 ±0.9	-1.834	-6.362	0.001
**C18:3n3**	0.1 ±0.1	0.5 ±0.1	0.323	12.263	0.000	0.4 ±0.1	0.245	9.498	0.000
**C18:4n3**	14.3 ±3.1	18.9 ±4.3	4.224	3.898	0.008	22.1 ±5.9	7.835	7.267	0.000
**C20:0**	0.3 ±0.1	0.3 ±0.1	-0.041	-2.034	0.089	0.3 ±0.1	-0.077	-3.893	0.009
**C20:1n9**	1.8 ±1.4	1.6 ±1.4	-0.152	-0.384	0.702	1.6 ±1.7	-0.130	-0.336	0.737
**C20:2**	0.3 ±0.1	0.4 ±0.3	0.097	1.788	0.124	0.3 ±0.1	-0.001	-0.011	0.992
**C20:3n6**	1.5 ±0.6	1.3 ±0.4	-0.280	-2.275	0.069	1 ±0.5	-0.664	-5.457	0.003
**C20:4n6**	4.8 ±1.5	4.7 ±1.4	-0.245	-0.511	0.629	3.9 ±0.9	-1.161	-2.429	0.054
**C20:4n3**	0.7 ±0.3	0.7 ±0.3	-0.018	-0.351	0.727	0.6 ±0.3	-0.165	-3.217	0.002
**C20:5n3**	2.6 ±0.9	2.7 ±0.7	0.122	1.169	0.246	2.9 ±0.8	0.353	3.421	0.001
**C22:0**	0.1 ±0.1	0.2 ±0.1	0.044	2.802	0.031	0.2 ±0	0.021	1.340	0.230
**C22:1n9**	0.4 ±0.2	0.5 ±0.2	0.005	0.101	0.923	0.5 ±0.2	-0.020	-0.372	0.723
**C23:0**	0.2 ±0.1	0.2 ±0.1	0.015	0.530	0.597	0.2 ±0.1	-0.032	-1.143	0.256
**C22:4n6**	3.3 ±1	3.2 ±1	-0.108	-0.392	0.710	3 ±0.6	-0.343	-1.253	0.262
**C22:5n3**	0.6 ±0.2	0.7 ±0.2	-0.008	-0.232	0.817	0.7 ±0.2	-0.022	-0.665	0.508
**C22:6n3**	8.9 ±1.9	10.3 ±2	1.341	3.122	0.018	10.4 ±2.1	1.142	2.697	0.035
**^{15}N (‰)**	-1.49 ±0.41	0.61 ±0.6	2.188	10.731	0.000	1.95 ±0.56	3.501	17.316	0.000
**^{13}C (‰)**	-15.98 ±0.8	-16.05 ±0.75	0.009	0.041	0.967	-16.22 ±0.98	-0.128	-0.612	0.542
**C:N**	5.79 ±0.36	5.48 ±0.29	-0.250	-2.775	0.035	5.16 ±0.37	-0.608	-6.807	0.001

Supplementary Table 5. Symbiont biomarker data summary and mixed effects linear model results. Table shows fatty acid (% total), isotope (permill) and elemental ratio summary data (mean and standard deviation, control: n = 30, F_2x: n = 25, F_6x: n = 32) with associated coefficient model estimates ('estimate'), F statistic ('statistic') and p value from linear mixed effects models.

biomarker	autotrophic	heterotrophic	statistic.W	p.value
**12:0**	0.28± 0.27	0.05± 0.01	117.5	0
**14:0**	5.4± 1.16	1.81± 0.4	120	0
**14:1**	0.51± 0.14	0.01± 0.01	120	0
**16:0**	23.07± 2	17.23± 3.06	112	0.01
**16:1n9**	0.94± 0.49	0.49± 0.33	96	0.06
**16:1n7**	7.4± 1.7	2.34± 0.19	120	0
**16:2**	0.56± 0.22	0.5± 0.34	52.5	0.71
**18:0**	6.37± 1.45	8.51± 0.54	9	0.01
**18:1n9**	7.22± 1.96	18.07± 0.96	0	0
**18:1n7**	1.24± 0.4	9.32± 0.66	0	0
**18:2n6**	1.6± 0.25	7.74± 1.87	0	0
**18:3n6**	5.48± 1.17	0.25± 0.04	120	0
**18:3n3**	0.14± 0.08	17.48± 2.23	0	0
**18:4n3**	14.27± 3.1	1.77± 0.36	120	0
**20:0**	0.29± 0.1	0.29± 0.06	58.5	0.96
**20:1n9**	1.81± 1.35	0.54± 0.11	116	0
**20:2**	0.3± 0.08	0.4± 0.04	19	0.03
**20:3n6**	1.51± 0.56	0.18± 0.02	120	0
**20:4n6**	4.77± 1.5	2.92± 0.7	105	0.02
**20:4n3**	0.69± 0.28	0.38± 0.04	106	0.01
**20:5n3**	2.59± 0.86	5.42± 1.58	5	0
**22:0**	0.12± 0.05	0.64± 0.1	0	0
**22:1n9**	0.43± 0.19	0.09± 0.03	119	0
**23:0**	0.2± 0.12	0.02± 0.01	120	0
**22:4n6**	3.27± 1.03	0.15± 0.25	120	0
**22:5n3**	0.65± 0.2	0.14± 0.08	120	0
**22:6n3**	8.88± 1.9	3.31± 0.94	120	0
**^{15}N (‰)**	-1.49± 0.41	9.81± 1.04	0	0
**^{13}C (‰)**	-15.98± 0.8	-20.69± 0.89	120	0
**C:N**	5.79± 0.36	4.48± 0.14	120	0

Supplementary Table 6. Summary statistics and Wilcoxon rank sum test results for experimental nutritional source groups. Table shows mean and standard deviation fatty acid data (% total), isotope ratios (permill) and one elemental ratio (no units) of both autotrophic (control coral symbionts, n = 31) and heterotrophic (nauplii, n = 4) nutritional source groups during the experiment. Wilcoxon rank sum test results show W statistic and p value for each biomarker.

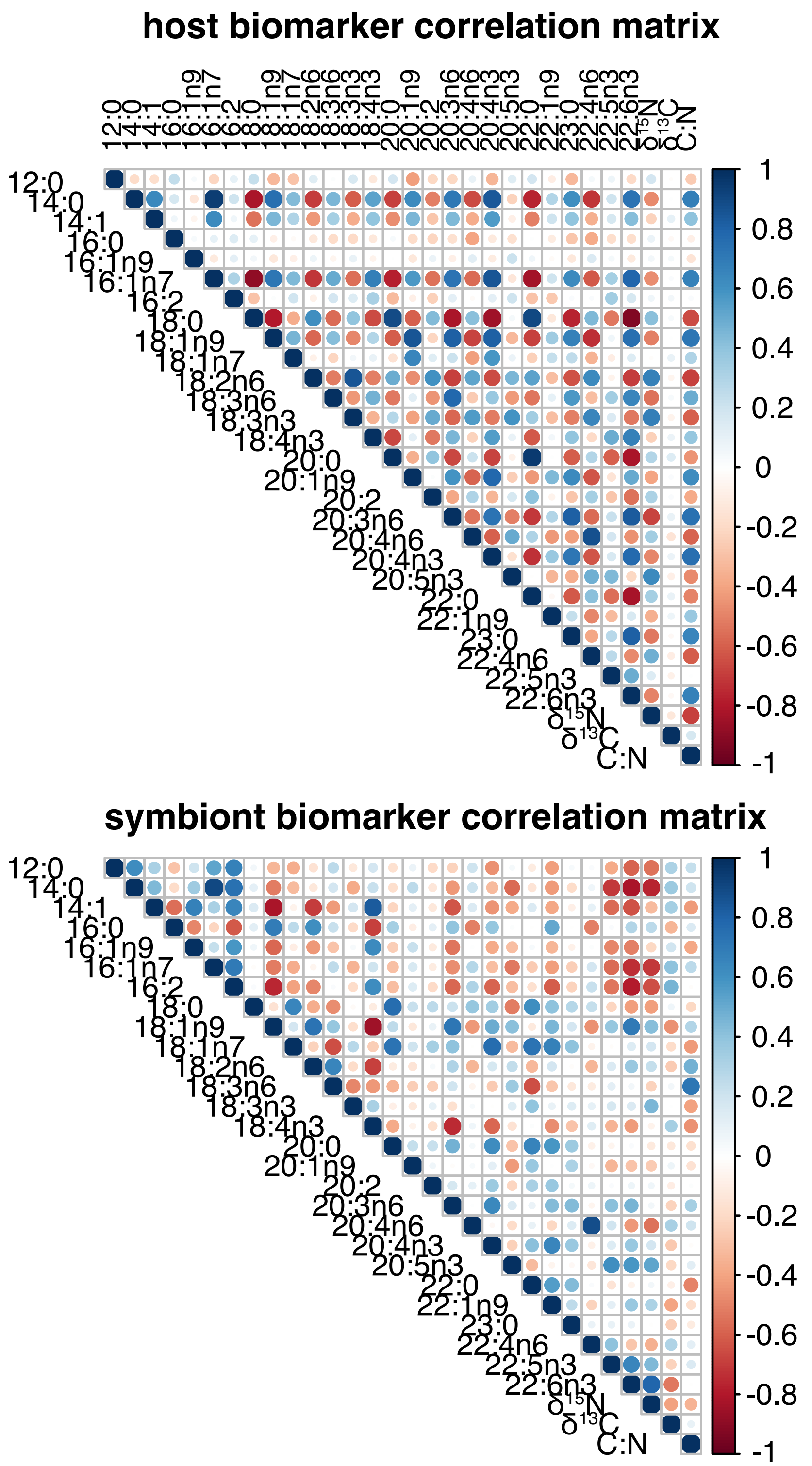

Supplementary Figure 2. Global correlation matrix of entire feeding experiment of FA biomarkers (% total) and elemental biomarkers for the host and symbiont fraction for the entire feeding experiment. Correlation values (R) are colored by a gradient, with positive correlations in blue, and negative correlations in red, darkness of color indicates strength of correlation, with transparent color indicating ~ no correlation between the two fatty acids.

parameter	control	F_2x	F_6x	B_F_6x
**% N host tissue**	1.62	1.74	2.18	1.58
**mg host tissue cm^{-2}**	10.34	11.2	12.14	8.78
**µg host N cm^{-2}**	156.88	185.63	232.87	132.71
**cumulative nauplii eaten cm^{-2}**	NA	918.04	1996.81	1609.64
**µg N consumed cm^{-2}**	NA	253.82	552.08	445.03
**% host N turnover**	~ 0	22.45	38.09	33.59
**N turnover potential**	NA	1.37	2.37	3.35
**% heterotrophic N integrated**	NA	16.42	16.07	<10.02

Supplementary Table 7. Treatment averaged soft tissue nitrogen data with associated nitrogen mass consumption data. 'N turnover potential' represents the number of times the coral host could have completely turned over its nitrogen pool with heterotrophic nitrogen.

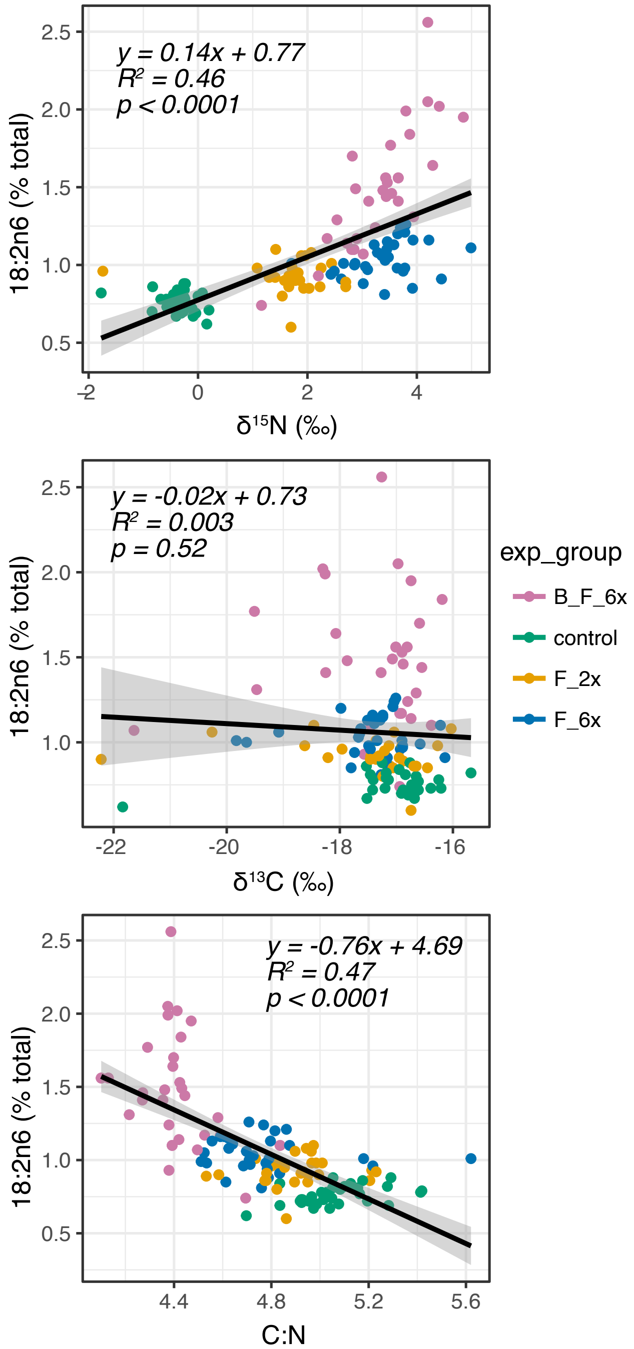

Supplementary Figure 3. Essential fatty acid 18:2n6 plotted against isotope and elemental ratios. Data in each panel are plotted with an associated linear model (black line) with a 95% confidence interval (shaded region around the black line). Top panel shows 18:2n6 plotted against nitrogen, middle panel shows 18:2n6 plotted against carbon isotope ratio, bottom panel shows 18:2n6 plotted against carbon to nitrogen ratio.

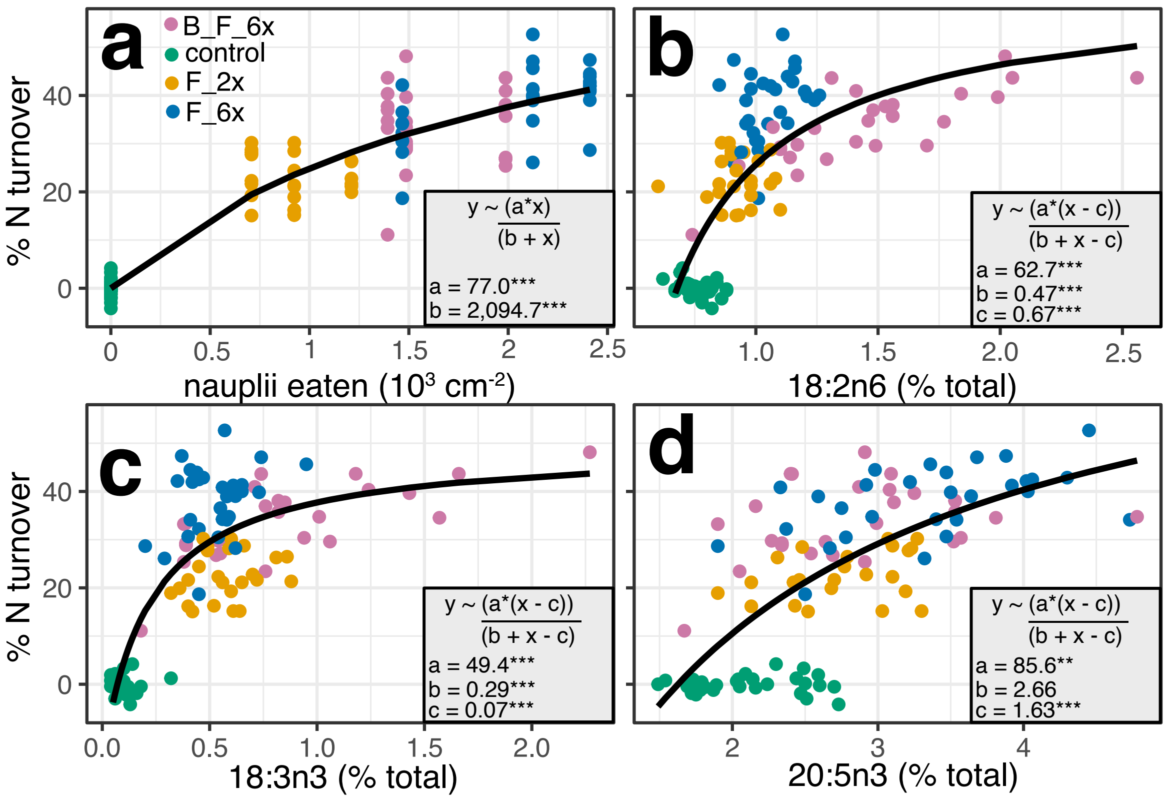

Supplementary Figure 4. Multiplot of host nitrogen turnover with heterotrophically acquired nitrogen and various host tissue parameters. **a**) Nitrogen turnover versus cumulative nauplii consumed over the entire experiment. Figure **a** was fitted to a Michaelis-Menten hyperbolic equation with a full zero intercept while figures **b**, **c**, and **d** were fitted to a modified Michaelis-Menten equation in which the fitted line does not go through the origin, with the offset on the x-axis represented by parameter 'c'. Fitted parameter estimates are listed on each plot with associated significance level of each fitted parameter ($p < 0.05$ = *; $p < 0.01$; = **; $p < 0.001$ = ***).

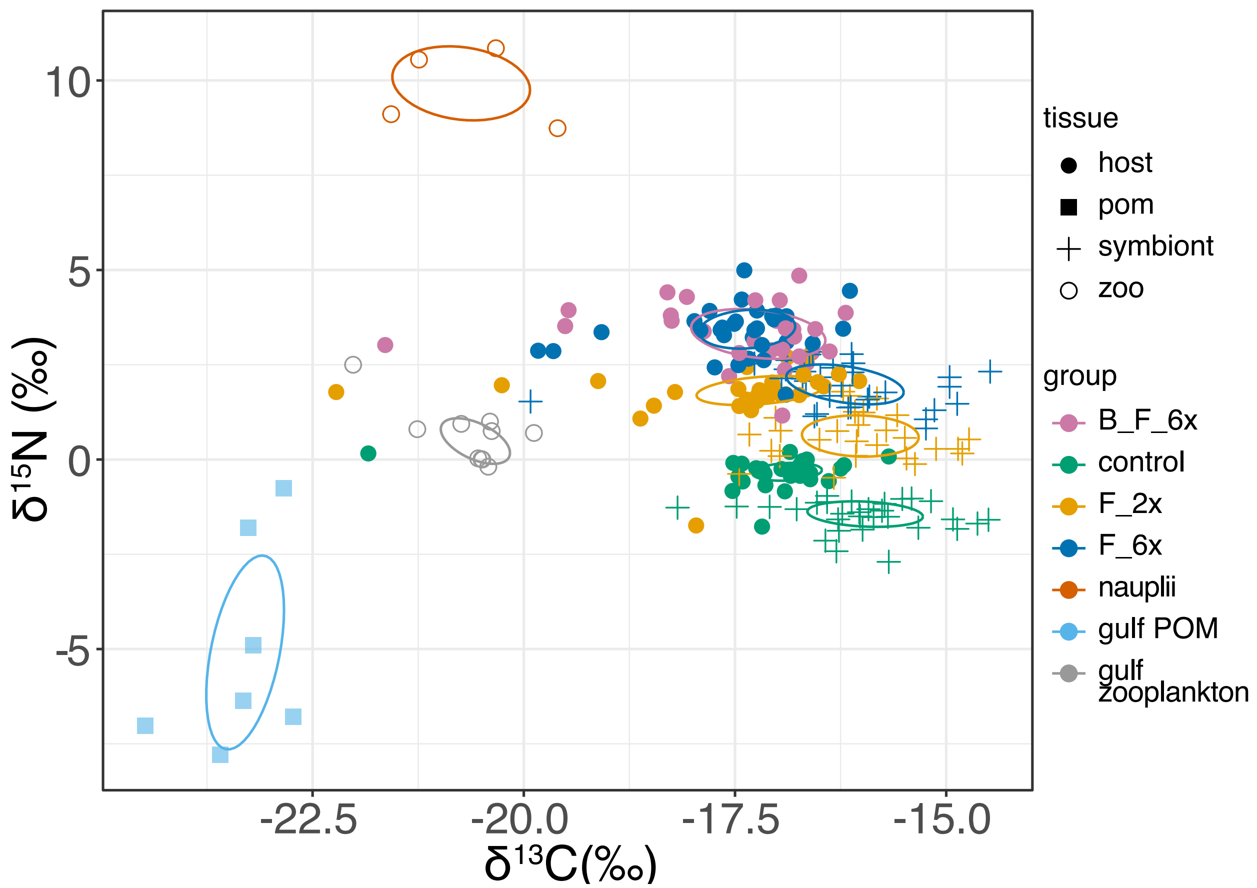

Supplementary Figure 5. Isotope biplot of coral host, coral symbiont, *Artemia* nauplii, and *in situ* zooplankton and particulate organic matter (POM) sampled from the Gulf of Aqaba. This plot shows all isotope data grouped by tissue type (coral host, coral symbiont, zooplankton and particulate organic matter) and includes experimental groups (coral host, coral symbiont, and nauplii (heterotrophic source during experiment)) and environmental samples from the coral collection site (gulf POM and gulf zooplankton) with 40% confidence ellipses. Experimental coral groups are grouped by treatment type: control (unfed), F_2x (fed two times week^{-1}), F_6x (fed six times week^{-1}) and B_F_6x (bleached and fed six times week^{-1}).

	Start temp. (°C)	Hold time (min)	Ramp rate (°C min^{-1})	Final temp (°C)	Final Hold time (min)
Ramp 1	50	1	10	150	0
Ramp 2	150	0	4	265	0

Supplementary Table 8. Gas Chromatograph oven temperature ramp profile.

	Inlet (He)	Septum Purge He)	Split vent (He)	Column Flow (He)	FID (air)	FID (N_2)	FID (H_2)
Gas Flow (mL min^{-1})	60	3.5	54	2.5	475	60	120

Supplementary Table 9. Gas Chromatograph Flame Ionization Detector gas flow rates.
