# Supplementary material for: Selective nutrient incorporation may underestimate heterotrophy of a mixotrophic reef-building coral
Source: Commun Biol. 2025 Aug 26;8:1285. doi: 10.1038/s42003-025-08621-8 (PMC12381372; doi:10.1038/s42003-025-08621-8)
Supplement: Supplementary file 3 — Reporting Summary [file 42003_2025_8621_MOESM3_ESM.pdf]

nature portfolio

Corresponding author(s): Connor Love

Last updated by author(s): Jul 14, 2025

Reporting Summary

Nature Portfolio wishes to improve the reproducibility of the work that we publish. This form provides structure for consistency and transparency in reporting. For further information on Nature Portfolio policies, see our Editorial Policies and the Editorial Policy Checklist.

Statistics

For all statistical analyses, confirm that the following items are present in the figure legend, table legend, main text, or Methods section.

n/a	Confirmed	
☐	☒	The exact sample size (*n*) for each experimental group/condition, given as a discrete number and unit of measurement
☐	☒	A statement on whether measurements were taken from distinct samples or whether the same sample was measured repeatedly
☐	☒	The statistical test(s) used AND whether they are one- or two-sided<br>*Only common tests should be described solely by name; describe more complex techniques in the Methods section.*
☐	☒	A description of all covariates tested
☐	☒	A description of any assumptions or corrections, such as tests of normality and adjustment for multiple comparisons
☐	☒	A full description of the statistical parameters including central tendency (e.g. means) or other basic estimates (e.g. regression coefficient) AND variation (e.g. standard deviation) or associated estimates of uncertainty (e.g. confidence intervals)
☐	☒	For null hypothesis testing, the test statistic (e.g. F, t, r) with confidence intervals, effect sizes, degrees of freedom and P value noted<br>*Give P values as exact values whenever suitable.*
☒	☐	For Bayesian analysis, information on the choice of priors and Markov chain Monte Carlo settings
☒	☐	For hierarchical and complex designs, identification of the appropriate level for tests and full reporting of outcomes
☒	☐	Estimates of effect sizes (e.g. Cohen's *d*, Pearson's *r*), indicating how they were calculated

Our web collection on statistics for biologists contains articles on many of the points above.

Software and code

Policy information about availability of computer code

Data collection	no software was used for data collection.
Data analysis	R (version 4.2.1) and R studio (version 2022.12.0+353) for statistics<br>CHROMuLAN (v0.79) for GC-FID peak integration<br>OpenChrom (v1.5.1) for GC-MS analysis for compound identification<br>IsoDat (v3.0) for isotope analysis

For manuscripts utilizing custom algorithms or software that are central to the research but not yet described in published literature, software must be made available to editors and reviewers. We strongly encourage code deposition in a community repository (e.g. GitHub). See the Nature Portfolio guidelines for submitting code & software for further information.

Data

Policy information about availability of data

All manuscripts must include a data availability statement. This statement should provide the following information, where applicable:

- Accession codes, unique identifiers, or web links for publicly available datasets
- A description of any restrictions on data availability
- For clinical datasets or third party data, please ensure that the statement adheres to our policy

Data will be made publicly available at the time of publication

Research involving human participants, their data, or biological material

Policy information about studies with human participants or human data. See also policy information about sex, gender (identity/presentation), and sexual orientation and race, ethnicity and racism.

Reporting on sex and gender	NA
Reporting on race, ethnicity, or other socially relevant groupings	NA
Population characteristics	NA
Recruitment	NA
Ethics oversight	NA

Note that full information on the approval of the study protocol must also be provided in the manuscript.

Field-specific reporting

Please select the one below that is the best fit for your research. If you are not sure, read the appropriate sections before making your selection.

☐ Life sciences ☐ Behavioural & social sciences ☒ Ecological, evolutionary & environmental sciences

For a reference copy of the document with all sections, see nature.com/documents/nr-reporting-summary-flat.pdf

Ecological, evolutionary & environmental sciences study design

All studies must disclose on these points even when the disclosure is negative.

Study description	The study was a controlled feeding experiment with the coral Stylophora pistillata. Corals were collected from an underwater nursery and acclimated to aquarium tanks for roughly one week. There were 10 fragments collected from 12 colonies and randomly distributed among tanks and treatments. There were 3 aquarium tanks per treatment with ~10 fragments per tank (n = 30 fragments per treatment). There were 4 treatments including: control (unfed, unaltered), fed 2x per week, fed 6x per week, and bleached and fed 6x a week. Corals were then subjected to experimental treatments for ~3 weeks and feeding measurements and non-destructive phsyiological measurements were taken. At the end of the experiment corals were flash frozen and processed for biological tissues. Each coral fragment was separated into component host and symbiont fractions and fatty acid, isotope and physiological measurements were taken on each fraction. Coral food (Artemia nauplii) and reef collected zooplankton and particulate organic matter were also subjected to fatty acid and isotope analysis.
Research sample	The reef-building coral Stylophora pistillata was chosen since it is well studied and is a common reef-building coral found in the Red Sea and many other reefs around the world.
Sampling strategy	No sample size calculation was made. During experimental design a sufficiently large sample size (n = 30 per treatment) was made so as to ensure there was more than enough data for relatively straightforward statistical tests.
Data collection	Feeding data was collected by counting the concentration of Artemia nauplii before and after feeding (collected by Connor Love). Non-destructive physiological measurements (skeltal growth and photo physiology) were collected by the buoyant weight method and by subjecting dark acclimated corals to imaging PAM fluorometer, respectively (collected by Connor Love). Tissue physiological measurements: protein, chlorophyll, symbiont density and surface area were collected by a Bradford assay, acetone extraction, counting with a microscope and wax dipping coral fragments (collected by Marleen Stuhr). Fatty acid data and isotope ratio measurements were done by gas chromatography flame ionization detector and by isotope ratio mass spectrometer coupled to an elemental analyzer (collected by Connor Love).
Timing and spatial scale	The experiment started 11/30/2019 and ended on 12/22/2019. Feeding measurements were taken nearly daily (6 our of 7 days of

Timing and spatial scale	the week) and buoyant weight and PAM measurements were taken roughly every 6 days. All tissue analysis was done after experimentation.
Data exclusions	no data was excluded from analysis.
Reproducibility	Reproduction of experiments was not possible due to time constraints, resources and personnel.
Randomization	Coral fragments were randomly assigned to treatment groups by using a random number generator.
Blinding	Blinding was not possible for our study because the people performing data acquisition were also those involved in collection and experimental design.

Did the study involve field work? ☒ Yes ☐ No

Field work, collection and transport

Field conditions	Field work was conducted in the Gulf of Aqaba in the northern Red Sea only for fragment collection. Gulf of Aqaba is a tropical reef ecosystem governed by temperatures from 20-30 C depending on season.
Location	Samples were taken at the underwater nursery at ~ 5-8 m depth; N 29.50000° E 34.93333°.
Access & import/export	Habitats were accessed via SCUBA and samples were collected from a catalog of corals grown and owned by the Inter-University Institute for Marine Sciences. Tissues were freeze-dried for transportation.
Disturbance	Coral fragment collection was completed by quickly cutting colonies with clippers. Divers ensured to not touch any other corals, remain off the bottom and stayed in the water for no longer than was necessary.

Reporting for specific materials, systems and methods

We require information from authors about some types of materials, experimental systems and methods used in many studies. Here, indicate whether each material, system or method listed is relevant to your study. If you are not sure if a list item applies to your research, read the appropriate section before selecting a response.

Materials & experimental systems

n/a	Involved in the study
☒	☐ Antibodies
☒	☐ Eukaryotic cell lines
☒	☐ Palaeontology and archaeology
☐	☒ Animals and other organisms
☒	☐ Clinical data
☒	☐ Dual use research of concern
☒	☐ Plants

Methods

n/a	Involved in the study
☒	☐ ChIP-seq
☒	☐ Flow cytometry
☒	☐ MRI-based neuroimaging

Animals and other research organisms

Policy information about studies involving animals; ARRIVE guidelines recommended for reporting animal research, and Sex and Gender in Research

Laboratory animals	study did not involve laboratory animals.
Wild animals	Fragments of Stylophora pistillata were collected from a nursery in the Gulf of Aqaba owned by the Inter-University Institute for Marine Sciences. Fragments were subjected to experimental treatment in aquarium tanks and were flash frozen at the end of experiment for tissue processing.
Reporting on sex	Does not apply.
Field-collected samples	Coral fragments collected were kept in 40 L aquarium with filtered seawater at ambient Gulf of Aqaba temperature.
Ethics oversight	No ethical approval or guidance was required since samples were collected from the Interuniversity Institute for Marine Sciences underwater nursery in which corals are grown on a metal scaffolding within the Gulf of Aqaba specifically for research purposes and to minimize impacts of coral collection from the existing reef.

Note that full information on the approval of the study protocol must also be provided in the manuscript.

Plants

Seed stocks	NA
Novel plant genotypes	NA
Authentication	NA
